# Imaging modality for measuring the presence and extent of the labral lesions of the shoulder: a systematic review and meta-analysis

**DOI:** 10.1186/s12891-019-2876-6

**Published:** 2019-10-27

**Authors:** Fanxiao Liu, Xiangyun Cheng, Jinlei Dong, Dongsheng Zhou, Qian Sun, Xiaohui Bai, Dawei Wang

**Affiliations:** 1 Department of Orthopaedics, Shandong Provincial Hospital affiliated to Shandong University, No.324, Road Jing Wu Wei Qi, Jinan, 250021 Shandong China; 2 Department of Orthopaedics, The 2nd Hospital of Shanxi Medical University, Taiyuan, Shanxi China; 3 Department of General and Paediatric Surgery, Yantai Yuhuangding Hospital affiliated Qingdao University, Yuhuangding eastern road 20, Yantai, China; 4 Department of Clinical Laboratory, Shandong Provincial Hospital affiliated to Shandong University, Jing Wu Road 324, Jinan, 250021 Shandong China

**Keywords:** Labral lesions, MRI, MRA, CTA, Diagnostic value, Meta-analysis

## Abstract

**Background:**

Multiple published studies quantitatively analysing the diagnostic value of MRI, MR arthrography (MRA) and CT arthrography (CTA) for labral lesions of the shoulder have had inconsistent results. The aim of this meta-analysis was to systematically compare the diagnostic performance of MRI, MRA, CTA and CT.

**Methods:**

Two databases, PubMed and EMBASE, were used to retrieve studies targeting the accuracy of MRI, MRA, CTA and CT in detecting labral lesions of the shoulder. After carefully screening and excluding studies, the studies that met the inclusion criteria were used for a pooled analysis, including calculation of sensitivity and specificity with 95% confidence intervals (CIs) and the area under the hierarchical summary receiver operating characteristic (HSROC) curves.

**Results:**

The retrieval process identified 2633 studies, out of which two reviewers screened out all but 14 studies, involving a total of 1216 patients who were deemed eligible for inclusion in the meta-analysis. The results assessing the diagnostic performance of MRI vs. MRA for detecting labral lesions showed a pooled sensitivity of 0.77 (95% CI 0.70–0.84) vs. 0.92 (95% CI 0.84–0.96), a specificity of 0.95 (95% CI 0.85–0.98) vs. 0.98 (95% CI 0.91–0.99), and an area under the HSROC curve of 3.78 (95% CI 2.73–4.83) vs. 6.01 (95% CI 4.30–7.73), respectively.

**Conclusion:**

MRA was suggested for use in patients with chronic shoulder symptoms or a pathologic abnormality. MRI is by far the first choice recommendation for the detection of acute labral lesions. CT should be a necessary supplemental imaging technique when there is highly suspected glenoid bone damage.

## Background

The glenoid labrum, composed of fibro-cartilage, is a ring or band structure that effectively increases the depth of the glenoid fossa [1]. Lesions of the glenoid labrum, occurring with glenohumeral instability, result in serious shoulder pain because of the destruction of free nerve endings located in the peripheral part of the glenoid labrum and the subacromial bursae [2, 3].

Based on their location and lesion features in imaging [4], disorders of the glenoid labrum have been broadly categorized as superior, posterior, inferior or anterior lesions [5]. Specifically, superior labral anterior-posterior tears (SLAP), initially described by Andrews et al. in 1985, have been an ongoing diagnostic challenge in the clinic [6]. Additionally, Bankart lesions are one kind of injury on the anteroinferior aspect of the glenoid labral complex, which are thought to predispose shoulders to recurrent dislocation [7, 8]. The integrity of the labrum and whether any bone has been avulsed or missing from the bony glenoid determined the different treatment strategies. For example, SLAP lesions were usually managed by arthroscopy at present [9] while detached Labra is often treated by open surgical repair [10]. Because of the serious pain associated with these injuries and the limitations they place on participation in high-level activities, the need to evaluate accuracy, efficiency, and economics of diagnostic tests for labral damage is increasingly important [9]. In addition, reorganization of the integrity of the glenoid labrum is an essential factor for clinicians to consider when making treatment decisions (i.e., to use conservative vs. surgical strategies) [10]. Medical imaging technologies not only provide rich and useful information to support findings from the medical history and physical examination but also demonstrate the pathoanatomy of shoulder dysfunction of the shoulder [11]. Therefore, a suitable choice of imaging technique could help to establish an appropriate treatment strategy.

Many imaging methods, including arthrography, computed tomography arthrography (CTA), magnetic resonance imaging (MRI), direct MR arthrography (D-MRA) and indirect MR arthrography (I-MRA) have been used to image the glenoid labrum as well as the associated structures of the capsular mechanism [12]. Shoulder MRI is becoming quite popular as a screening examination for the detection of labral abnormalities [13]. However, intra-articular structures of the shoulder are not well imaged by MRI when insufficient fluid is present to outline the glenoid contour [14].

MRA of shoulders mainly included indirect shoulder magnetic resonance arthrography (I-MRA) and direct shoulder magnetic resonance arthrography (D-MRA). D-MRA, which involves intra-articular administration of contrast agent, has become an established imaging modality for assessing different types of labral lesions [14]. Additionally, an alternative and less invasive technique, I-MRA, were intravenously administered contrast enhances the joint space and indirectly produces an arthrographic effect [15]. MRA is considered to have higher accuracy than MRI in the detection of glenoid labral tears, but it is invasive [15]. CTA does not have advantages in the evaluation of soft tissue injuries such as labral damage over MRI and MRA; however, it was proven to have much higher diagnostic accuracy for detecting bony defects of the glenoid [16]. With the development of MRI technologies, the diagnostic sensitivity and specificity of 3-Tesla (T) MRI versus MRA for assessing labral abnormalities is controversial to a certain extent [17]. A previous meta-analysis [18] suggested that MRA had greater diagnostic accuracy than MRI for the overall detection of glenoid labral lesions. The opposite result was obtained when diagnosing anterior glenoid labral lesions. Another meta-analysis demonstrated that MRA was superior to MRI for the detection of SLAP lesions [19]. A recent meta-analysis from 2018, involving 10 studies, revealed that 3.0 T MRA improved sensitivity for the diagnosis of anterior and posterior labral tears, but reduced specificity in the diagnosis of SLAP tears [20].

Recently, multiple high-quality studies [14, 21–23] were published, most of which used relatively high resolution for CTA and relatively high-field strength magnets and multidimensional imaging for MRI and MRA. Moreover, no studies have compared the diagnostic performance of MRI, D-MRA, I-MRA and CTA using side-by-side analysis in a single study for the detection of labral lesions. Therefore, an updated meta-analysis is warranted to determine if the new data and improved technology have had an impact on the diagnostic accuracy of a given pool of data.

The primary objective of this study was to perform a meta-analysis on the diagnostic accuracy of MRI, MRA, CTA and CT in the assessment of glenoid labral lesions. The second objective was to compare the diagnostic accuracy of MRI and MRA for detecting different types of labral lesions, such as anterior, posterior or superior lesions. The third objective was to evaluate the effect of magnet strength on the diagnostic accuracy of MRI and MRA for glenoid labral lesions.

## Methods

This meta-analysis was conducted based on the guidelines of the Preferred Reporting Items for a Systematic Review and Meta-analysis of Diagnostic Test Accuracy Studies (PRISMA-DTA) [24] statement. Patient informed consent and committee approval were not required for this study due to the use of published data.

### Selection and inclusion criteria

The keywords “MRI”, “magnetic resonance imaging”, “magnetic resonance arthrography”, “MR arthrography”, “MRA”, “computed tomography arthrography” “computed tomography”, “CT”, or “CTA” AND “labral” or “shoulder pain” were used to search two databases, PubMed and EMBASE, to retrieve published studies measuring the diagnostic accuracy of MRI, MRA, CT and CTA for labral lesions. The date of the newest search was November 1, 2018, and there was no language limitation. Additionally, a supplementary search by hand was further performed to screen the reference lists of the included studies.

The clinical trials that involved patients with labral lesions; assessing the diagnostic accuracy of MRI, MRA, CT and CTA for labral lesions and provided direct diagnostic data, including true-positive (TP), false-positive (FP), false-negative (FN) and true negative (TN), or data that enabled calculation of these parameters, met the inclusion criteria and were included in this meta-analysis. The study presenting the most data was included in this statistical analysis if any studies contained overlapping data. The review literature, no full-text studies, including conference summaries and meeting abstracts, or non-clinical studies, such as animal and cadaver experiments and biomechanics, were excluded.

### Data extraction and risk of bias

Each study found in the search process was screened, and its appropriateness for inclusion was determined. Information from each study were extracted into a standardized form independently by two blinded reviewers. The information included the following: the first author’s surname; year of publication; country of origin; basic information about the participants, such as number, age and sex; the main characteristics of the MRI, MRA and CTA and their analysis methods; and the original diagnostic data, including TP, FP, FN and TN outcome were extracted.

The risk of bias of each included study was measured utilizing a quality assessment tool (QUADAS-2), [25–27] which contains 11 items and is usually used for diagnostic accuracy studies.

### Statistical analysis

Two reviewers (Reviewers CXY and LFX) independently and blindly screened the search records from two databases, identified studies using the inclusion criteria, extracted the target data, and measured the quality of the studies using the aforementioned tool. Inconsistencies between reviewers were resolved by consensus.

The primary outcome of this meta-analysis was to compare the diagnostic value of MRI, MRA and CTA for labral lesions simultaneously in the included studies. To derive summary estimates of the diagnostic value of each modality, a bivariate random-effects model was applied to analyze the following pooled outcome estimates: sensitivity, specificity and hierarchical summary receiver operating characteristic (HSROC) [28, 29] curves based on the diagnostic data extracted from each included study. HSROC curves provide a 95% confidence interval (CI) and prediction regions. The secondary outcomes were the various subgroups (type of lesions) to determine the reliability of imaging techniques in the various subgroups. According to the PRISMA-DTA [24], the publication bias Deeks’ funnel plots [30] was omitted. All statistical analyses were calculated utilizing Stata v-12.0 and Meta-Disc v-1.4.

## Results

### Selection process

**Fig. 1:**
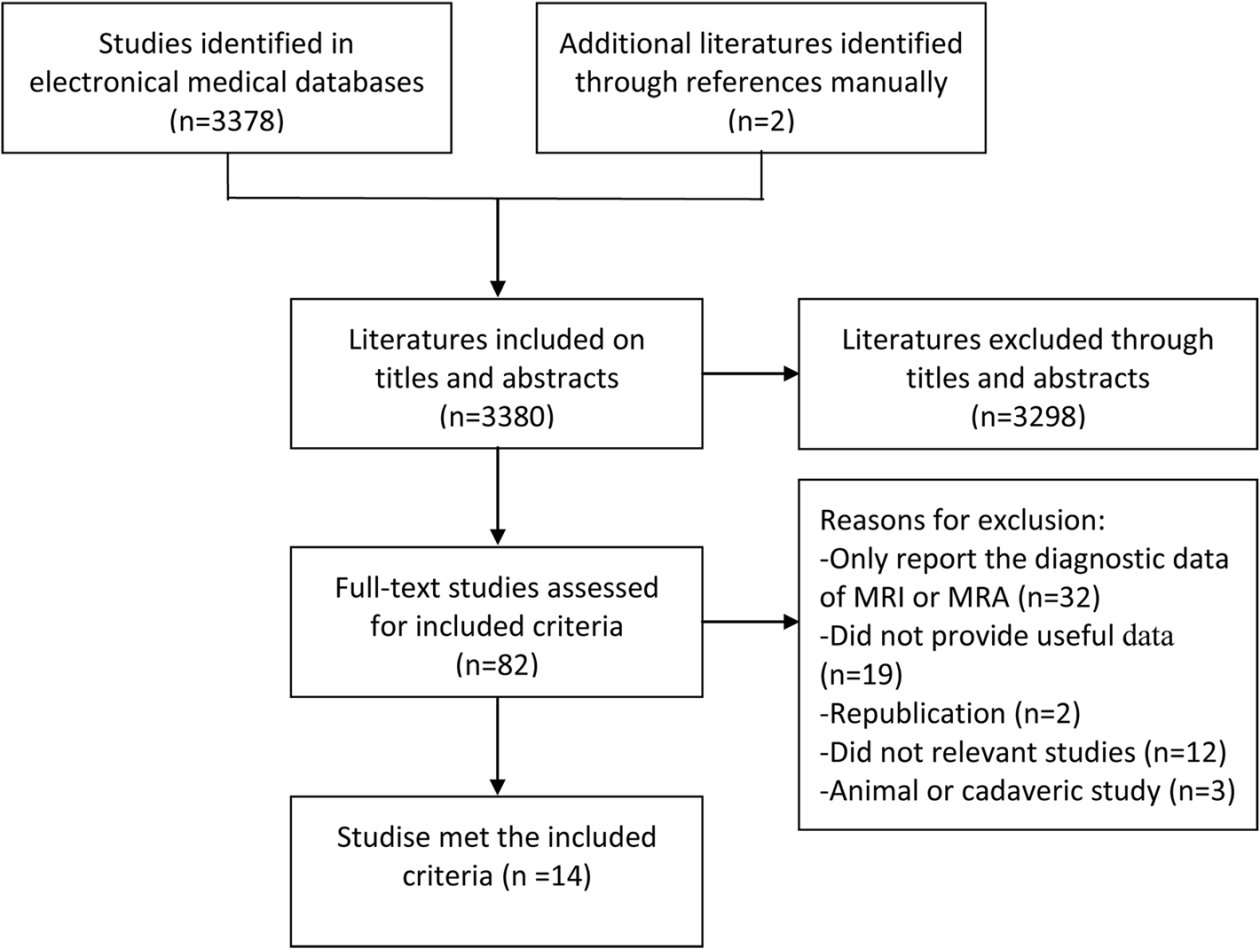
Selection flow chart for included studies in the meta-analysis

The initial search of the two chosen electronic databases and the subsequent screening process of potential studies is represented in Fig. 1. Of 2633 records identified during the database and bibliography searches, 1046 ineligible records were excluded due to repetition, and 1530 were excluded by screening titles and abstracts. Subsequently, further exclusions were performed by downloading and reviewing the full-text versions of the remaining studies. After a detailed search and selection process, 14 studies [15, 17, 21–23, 31–39] involving 1216 patients with labral lesions met the inclusion criteria for the meta-analysis.

### Study characteristics and quality assessment

Table 1 and Table 2 present the main characteristics of the participants and the included studies. All included articles were published between 1990 and 2016, with sample sizes ranging from 23 to 444 patients. A total of 14 studies [15, 17, 21–23, 31–39] used MRI and MRA to assess labral lesions. For all included studies, the gold standard for diagnosing labral lesions was arthroscopy or surgery. The methodological quality resulted in one study [35] receiving a score of 8, two studies [21, 32] receiving a score of 9, and the remaining 11 studies [15, 17, 22, 23, 31, 33, 34, 36–39] achieving an overall score of 10, according to the QUADAS-2 tool. The main characteristics of three imaging methods, MRI, MRA and CTA, are presented in Table 3.

**Table 1:** Main characteristics of the subjects from included studies

Study, year	No. of patients	Age, years mean (range)	Sex (M/F)	No. of shoulders	Clinical indication of shoulder	Methods	Final diagnosis of included patients
Flannigan, et al. 1990 [31]	23	45 (16–68)	18/5	23	Shoulder pain	MRI/MRA	Labral tears
Chandnani, et al. 1993 [32]	30	27 (19–39)	NA	30	Shoulder pain	MRA/CTA	Labral tears
Sano, et al. 1996 [33]	47	24 (14–45)	39/8	47	Shoulder pain	MRA/CTA	Labral tears
Wallny, et al. 1998 [34]	28	43 (21–63)	19/9	28	Clinically suspected labral injuries	MRI/MRA	Labral tears
Herold, et al. 2003 [38]	35	47.3 (18–67)	26/9	35	Acute or chronic shoulder disorder	MRI/MRA	SLAP
Reuss, et al. 2006 [35]	83	NA	NA	83	Shoulder pain	MRI/MRA	SLAP
Dinauer, et al. 2007 [15]	104	40 (18–65)	82/22	104	Mechanical symptoms	MRI/MRA	SLAP
Magee, et al. 2009 [17]	150	31 (14–50)	109/41	150	Shoulder pain	MRI/MRA	SLAP/Posterior/Anterior
Major, et al. 2011 [36]	42	33	28/14	42	Shoulder pain	MRI/MRA	Superior/Posterior/Anterior
Fallahi, et al. 2013 [37]	91	35 (15–70)	74/17	91	Shoulder pain	MRI/MRA	Labral tears
Mahmoud, et al. 2013 [39]	31	21–70	24/7	31	Shoulder lesion	MRA/CTA	SLAP/Bankart
Moroder, et al. 2013 [23]	48	30.8 (20–78)	40/8	48	Anterior shoulder instability	MRI/CT	Glenoid defect (bony)
Sheridan, et al. 2015 [22]	444	49	271/173	444	Shoulder pain	MRI/MRA	SLAP
El-Liethy, et al. 2016 [21]	60	35 (14–55)	NA	60	Trauma, shoulder pain, dislocation	MRI/MRA	Labral tears

*NA* No available. *SLAP* Superior labrum anterior-to-posterior

**Table 2:** Main characteristics of the included studies

Author, year	Country	Inclusion interval	Study design	Gold standard	Time from MRI/MRA to gold standard, days, mean (range)	Blinding	No. of readers	Reader experience (years)
Flannigan, et al. 1990 [31]	USA	NA	P	Arthroscopy/Open Surgery	NA	Yes	2	NA
Chandnani, et al. 1993 [32]	USA	NA	P	Arthroscopy/Open Surgery	NA	Yes	2	2/4
Sano, et al. 1996 [33]	Japan	NA	R	Arthroscopy	NA	Yes	2	NA
Wallny, et al. 1998 [34]	Germany	NA	P	Arthroscopy/Open Surgery	NA	Yes	2	NA
Herold, et al. 2003 [38]	Germany	NA	P	Arthroscopy	60 (33–175)	Yes	2	7/12
Reuss, et al. 2006 [35]	USA	09.1998–03.2003	R	Arthroscopy	NA	Yes	2	NA
Dinauer, et al. 2007 [15]	USA	09.2011–10.2030	P	Arthroscopy/Open Surgery	1–175	Yes	2	5
Magee, et al. 2009 [17]	USA	01.2007–07.2007	R	Arthroscopy	NA	Yes	2	10
Major, et al. 2011 [36]	USA	01.2007–07.2006	P	Arthroscopy	Less 3 months	Yes	3	30/15/6
Fallahi, et al. 2013 [37]	UK	01.2009–12.2011	R	Arthroscopy/Open Surgery	NA	Yes	2	14/6
Mahmoud, et al. 2013 [39]	Egypt	03.2011–05.2012	P	Arthroscopy	Less 100	Yes	2	NA
Moroder, et al. 2013 [23]	Austria	2006–2009	R	Arthroscopy/Open Surgery	NA	Yes	NA	NA
Sheridan, et al. 2015 [22]	USA	2006–2008	R	Arthroscopy/Open Surgery	NA	Yes	NA	NA
El-Liethy, et al. 2016 [21]	Egypt	06.2015–12.2015	R	Arthroscopy	NA	Yes	2	NA

*NA* No available, *R* Retrospective, *P* Prospective

**Table 3:** Main characteristics of MRI, MRA and CTA

Author, year	Scanner (MRI /MRA)			Method (MRA)	Technical parameters (MRI /MRA)				Analyzed image plane
	Vendor	Model	Magnetic strength/CT Slice		Sequence (MRI)	Sequence (MRA)	Slice thickness (mm)	NO. of analyzed image plane	
Flannigan, et al. 1990 [31]	GE Healthcare	Signa	1.5 T	Direct	T1WI (SE)	T1WI (SE)	4/4	1/1	Coronal
Chandnani, et al. 1993 [32]	NA	NA	1.5 T	Direct	T1WI SE pulse sequence	PDWI, T2WI (SE)	3/3	2/2	Axial, obl cor/Axial, obl cor
Sano, et al. 1996 [33]	Shimazu	NA	1.5 T	NA	NA	T1WI	2/4	3/3	Axial, obl cor, obl sag/Axial, obl cor, obl sag
Wallny, et al. 1998 [34]	Philips	ACS II	1.5 T	Indirect	T1WI,T2WI,PD	T1WI (FS)	3/3	2/2	Axial, obl cor/Axial, obl cor
Herold, et al. 2003 [38]	Siemens	Erlangen	1.5 T	Indirect	STIR, T1 SE,PD-T2 TSE,T1-Flash 2D,T1 SE	STIR, T1 SE,PD-T2 TSE,T1-Flash 2D,T2 SE	3/3	3/3	axial, parasag, paracor/Axial, parasag, paracor
Reuss, et al. 2006 [35]	NA	NA	1.5 T	Direct	NA	NA	NA	NA	NA
Dinauer, et al. 2007 [15]	GE healthcare	Signa	1.5 T	Indirect	T1WI (FSE, FS), T2WI (FSE, FS)	T1WI (FSE, FS)	3.5/3.5	3/3	Axial, obl cor, obl sag/Axial, obl cor, obl sag
Magee, et al. 2009 [17]	GE healthcare	Signa	3 T	Direct	T1WI (FSE),T2WI(FSE), T2WI (FSE, FS)	T1WI (FS)	4/4	3/3	Axial, obl cor, obl sag
Major, et al. 2011 [36]	Siemens	Signa	3 T	Direct	T1WI, T2 WI(FS), PDWI	T1WI (FS),T1WI, T2WI (FS)	3/3	4/4	Axial, obl cor, obl sag, sag
Fallahi, et al. 2013 [37]	Siemens	Avanto	1.5 T	Indirect	T1FS, PDFS, STIR, T2 GRE	T1FS, PDFS, STIR, T2 MEDIC	3/3	3/3	Para cor, sag, axial
Mahmoud, et al. 2013 [39]	Philips	Gyroscan NT	1.5 T/ 64-slice	Direct	NA	T1WI (FS), 3DWatSc, T2WI (SE)	3/2	3/2	Axial, obl cor, obl sag/Supine position, ABER
Moroder, et al. 2013 [23]	Siemens	Somatom sensation 64	1.5 T/ 64-slice	NA	At least two different sequences	NA	NA	3/3	Axial, parasag, paracor,3D reconstruction/Axial, parasag, paracor
Sheridan, et al. 2015 [22]	NA	NA	1.5 T	NA	PDWI, T2WI (FS)	T1WI (FS), T1WI(PD), T2WI (FSE)	NA	3/3	Axial, obl cor, sag/Axial, cor, sag
El-Liethy, et al. 2016 [21]	Philips&Simens	Gyroscan interna &Symphony	1.5 T	Direct	T1 (TSE), T2 (TSE), STIR(TSE), PD(TSE),GR (TSE)	T1FS (all pulse sequences)	NA	3/3	Axial, obl cor, obl sag /Axial, cor, sag

*TSE* Turbo spin echo, *GRE* Gradient echo, *PD* Proton density, *FS* Fat suppressed, *WI* Weighted image, *SPAIR* Spectral attenuated inversion recovery, *FSE* Fast spin-echo, *STIR* Short-TI inversion recovery, *SE* Spin echo, *Axi* Axial, *obl cor*, oblique coronal, *obl sag* oblique sagittal, *NR* not available, *3DWatSc* 3D-gradient echo images, *Para cor* paracoronal

### Diagnostic value of MRI and MRA (all labral lesions)

**Fig. 2:**
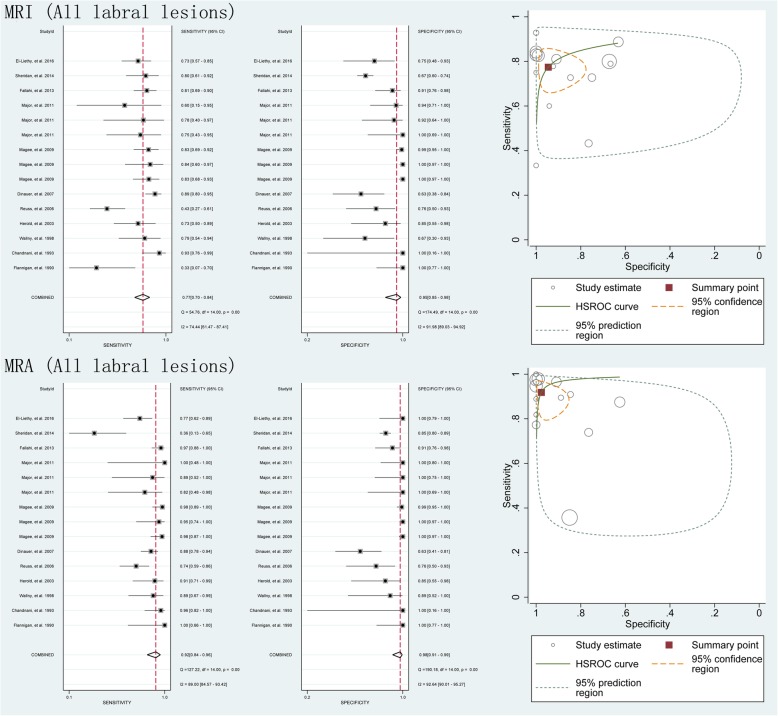
Pooled sensitivity, specificity and HSROC of MRI and MRA for detecting all labral lesions

The results comparing the diagnostic performance of MRI vs. MRA for detecting labral lesions in patients, as generated from the 7 studies [15, 17, 21, 22, 35–37] involving 1184 shoulders showed a pooled sensitivity of 0.77 (95% CI 0.70–0.84) vs. 0.92 (95% CI 0.84–0.96), a specificity of 0.95 (95% CI 0.85–0.98) vs. 0.98 (95% CI 0.91–0.99), and an area under the HSROC curve of 3.78 (95% CI 2.73–4.83) vs. 6.01 (95% CI 4.30–7.73), respectively (Fig. 2).

### Diagnostic value of D-MRA and I-MRA (all labral lesions)

**Fig. 3:**
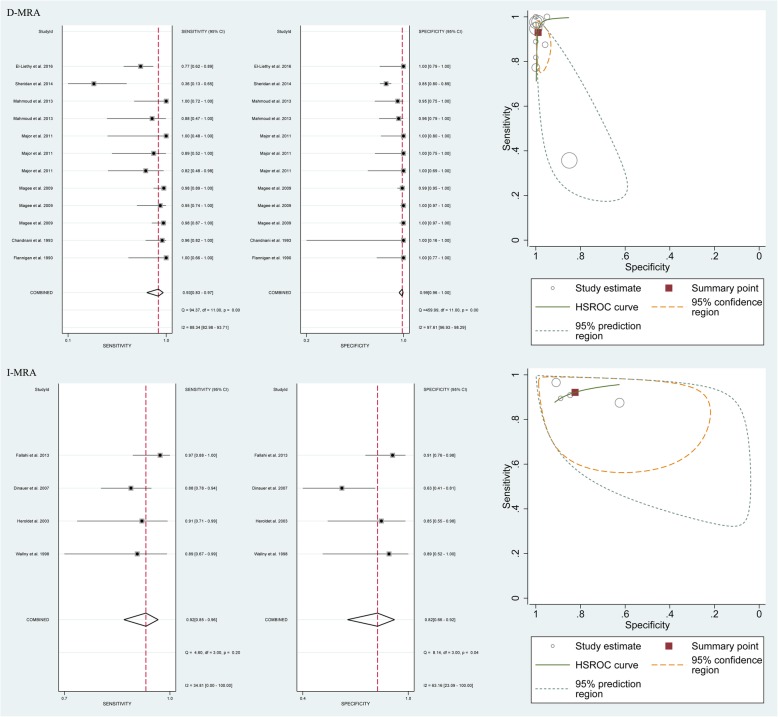
Pooled sensitivity, specificity and HSROC of D-MRI and I-MRA for detecting all labral lesions

The results comparing the diagnostic performance of D-MRI vs. I-MRA for detecting labral lesions showed a pooled sensitivity of 0.93 (95% CI 0.83–0.97) vs. 0.92 (95% CI 0.85–0.96), a specificity of 0.99 (95% CI 0.96–1.00) vs. 0.82 (95% CI 0.66–0.92), and an area under the HSROC curve of 7.20 (95% CI 5.25–9.16) vs. 4.37 (95% CI 2.36–6.39), respectively (Fig. 3).

### Diagnostic value of MRI and MRA (SLAP)

**Fig. 4:**
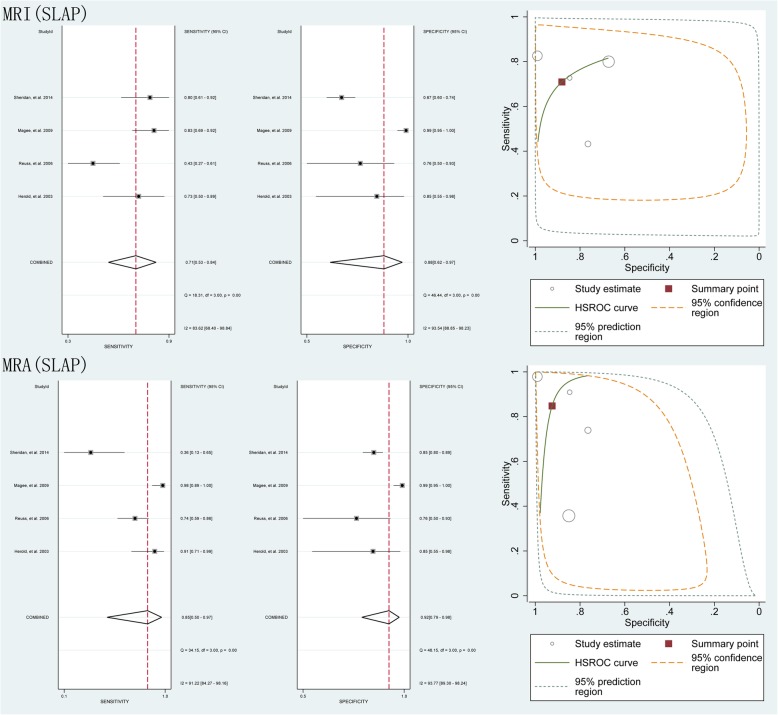
Pooled sensitivity, specificity and HSROC of MRI and MRA for detecting SLAP lesions

The results comparing MRI vs. MRA for detecting SLAP lesions, as generated from the 4 studies [17, 22, 35, 38] included in the present meta-analysis, involving 483 shoulders, demonstrated that the pooled results were as follows: the pooled sensitivity was 0.71 (95% CI 0.53–0.84) vs. 0.85 (95% CI 0.50–0.97), the specificity was 0.88 (95% CI 0.62–0.97) vs. 0.92 (95% CI 0.795–0.98), and the area under the HSROC curve was 2.67 (95% CI 0.86–4.48) vs. 4.62 (95% CI 1.29–7.95), respectively (Fig. 4).

**Fig. 5:**
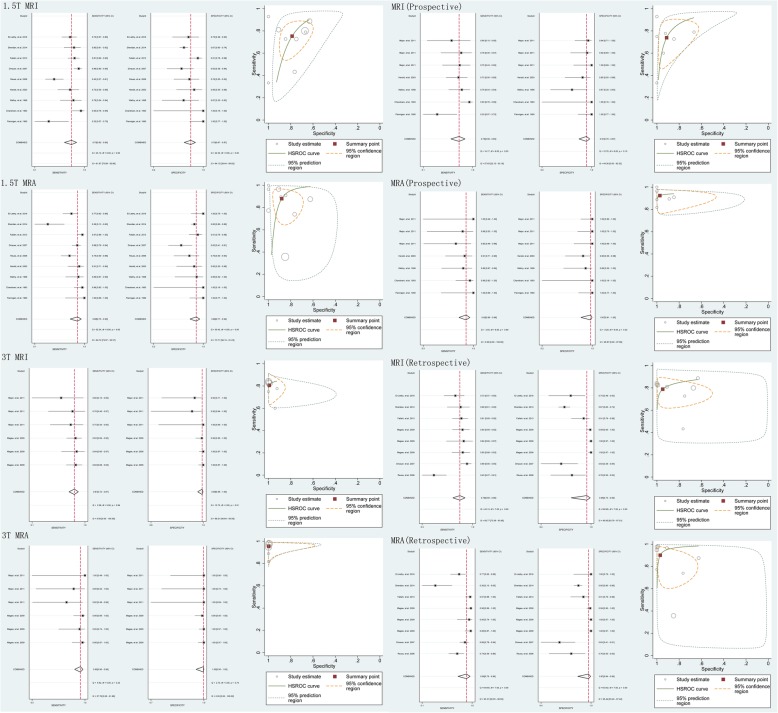
Pooled sensitivity, specificity and HSROC of 1.5 T MRI and MRA, 3 T MRI and MRA, MRI and MRA in prospective and retrospective design for detecting all labral lesions

The results of the subgroup analyses based on magnet strength (1.5-T and 3-T) and study type (prospective and retrospective), generated from the 9 studies [15, 21, 22, 31, 32, 34, 35, 37, 38] involving 668 shoulders, the 2 studies involving 516 shoulders, the 7 involving 182 shoulders and the 8 studies involving 1003 shoulders, all indicated that MRA had a higher accuracy than MRI in the detection of labral lesions (Fig. 5).

**Fig. 6:**
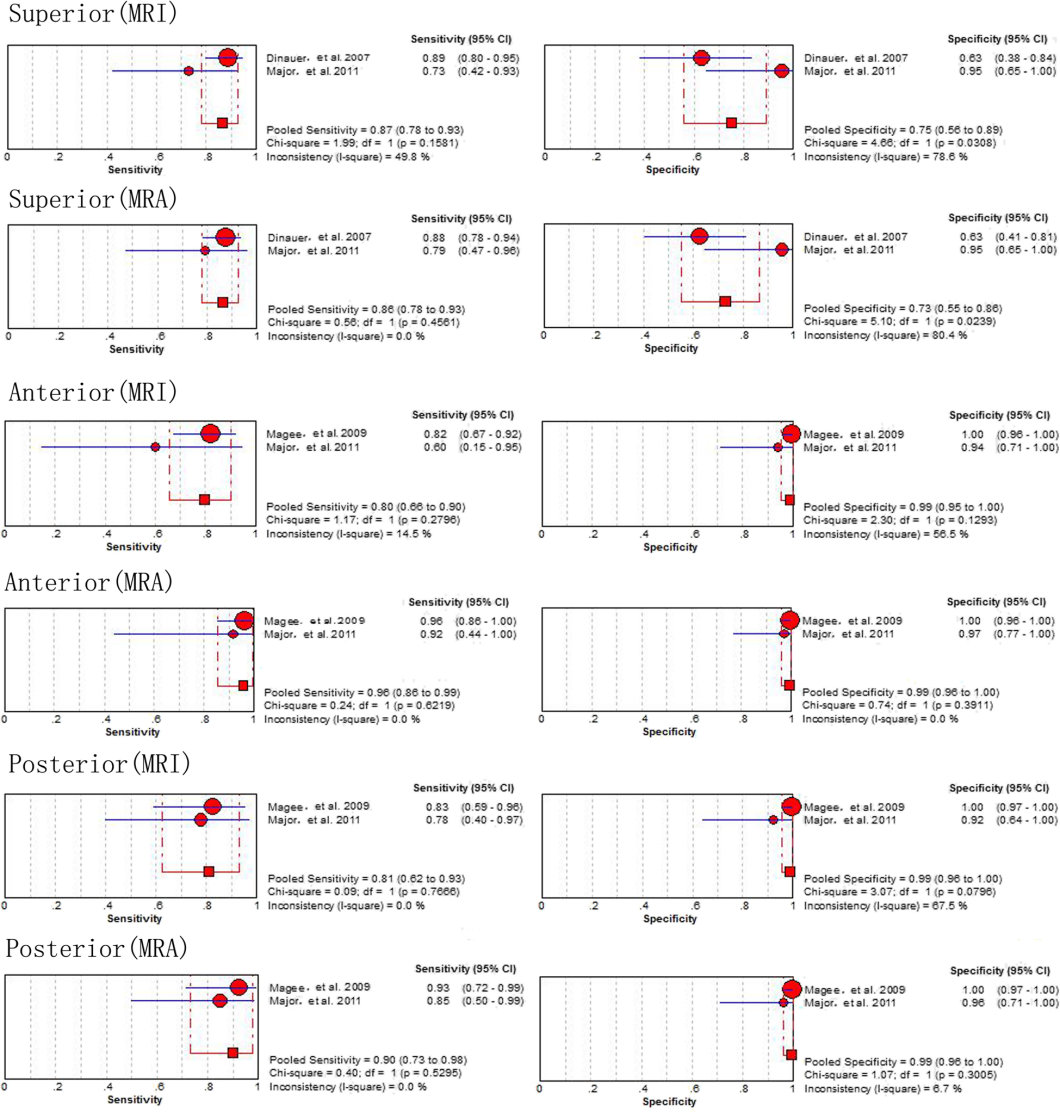
Pooled sensitivity and specificity of MRI and MRA for detecting superior, anterior and posterior labral lesions

The results of the subgroup analyses based on the location of labral lesions (posterior, anterior and superior) generated from the 2 studies [17, 36] involving 125 shoulders, the 2 studies [17, 36] involving 172 shoulders and the 2 studies [15, 36] involving 172 shoulders, demonstrated that MRA had a higher sensitivity and specificity than MRI **(**Fig. 6**)**.

### Diagnostic value of MRA and CTA

**Fig. 7:**
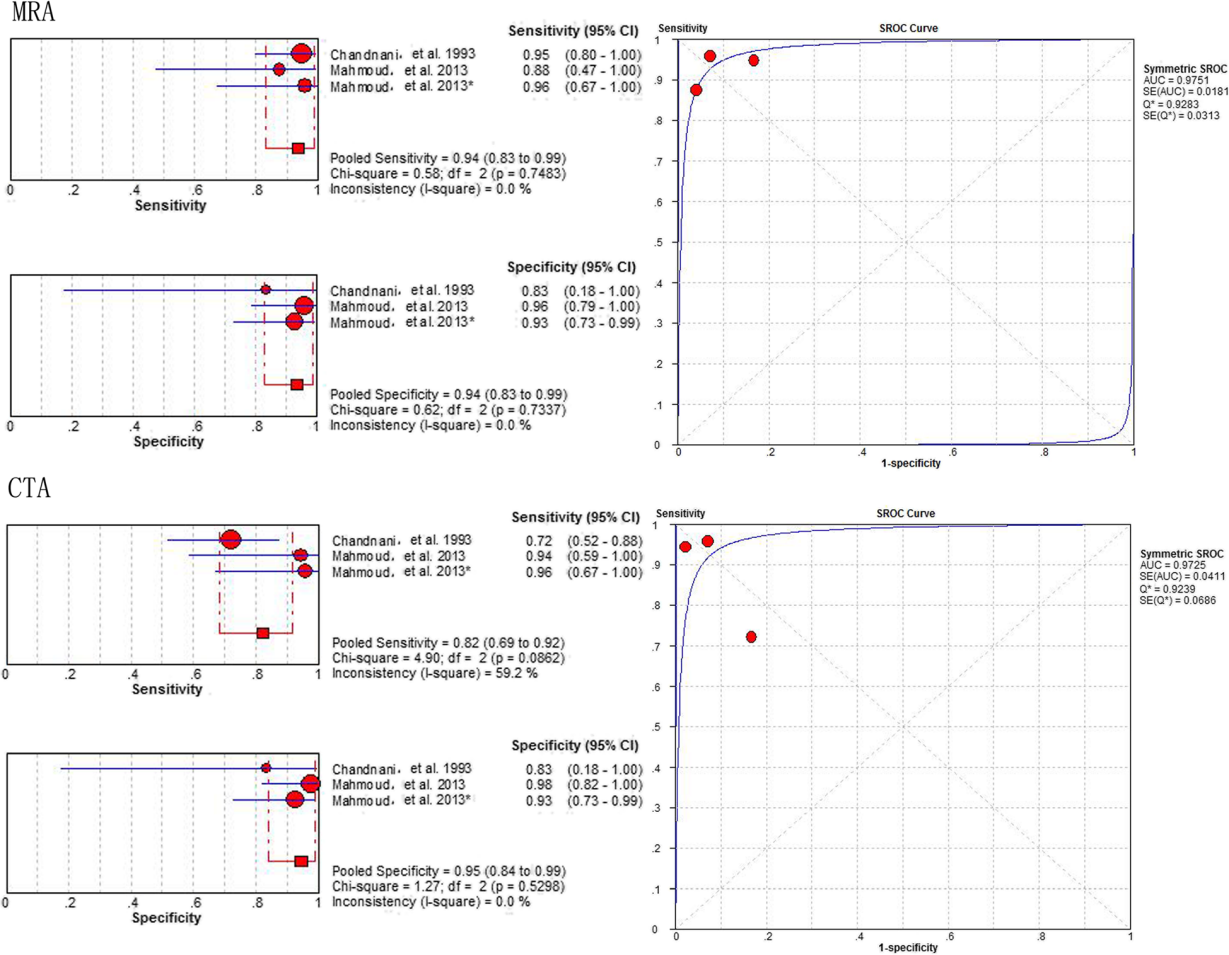
Pooled sensitivity, specificity and SROC of MRA and CTA for detecting all labral lesions

The results comparing the diagnostic performance of MRA vs. CTA for detecting labral lesions, generated from the 2 studies [32, 39], involving 93 shoulders, showed a pooled sensitivity of 0.94 (95% CI 0.83–0.99) vs. 0.82 (95% CI 0.69–0.92), a specificity of 0.94 (95% CI 0.83–0.99) vs. 0.95 (95% CI 0.84–0.99), and an area under the SROC curve of 0.9751(Q* = 0.9283) vs. 0.9725 (Q* = 0.9239), respectively (Fig. 7).

### Diagnostic value of MRI and CTA

**Fig. 8:**
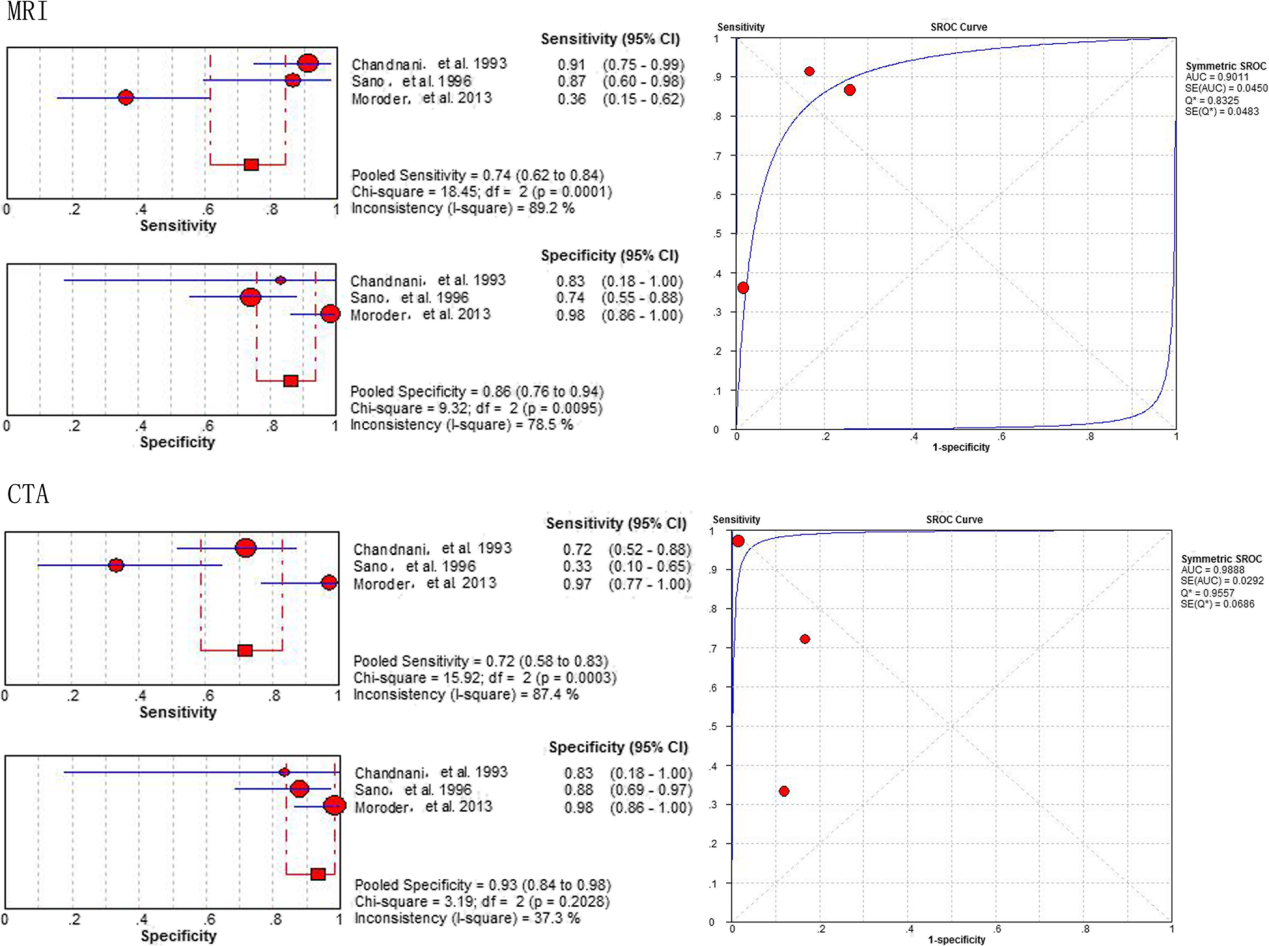
Pooled sensitivity, specificity and SROC of MRI and CTA for detecting all labral lesions

The diagnostic performance of MRI vs. CTA for detecting labral lesions in patients as generated from 3 studies [23, 32, 33], involving 124 shoulders, showed a pooled sensitivity of 0.74 (95% CI 0.62–0.84) vs. 0.72 (95% CI 0.58–0.83), a specificity of 0.86 (95% CI 0.76–0.94) vs. 0.93 (95% CI 0.84–0.98), and an area under the HSROC curve of 0.9011 (Q* = 0.8325) vs. 0.9888 (Q* = 0.9557), respectively (Fig. 8).

## Discussion

Lesions of the glenoid labrum are critical factors causing shoulder pain and disability [40, 41], which can seriously affect the quality of patients’ lives if without suitable diagnostic techniques and proper treatment strategies. The decision to perform arthroscopy or open surgery [42], as the ultimate treatment option of labral disorders, depends not only on the patients’ clinical histories and physical examinations but also on their imaging results [37], and accurate positioning of the tears undergoing surgery are largely affected by the pre-operative imaging reports [43]. Diagnostic accuracy and effective use of imaging technology are the main concerns of clinicians and patients. Therefore, it is essential to compare the accuracy of MRA (I-MRA and D-MRA), MRI and CTA for labral diagnosis and to analyse their advantages and disadvantages under various specific conditions.

It has long been important to address the roles of MRI and MRA as imaging tools for detecting pathologic labral lesions [15, 44]. While there is a large body of literature suggesting that MRA is superior to conventional MRI for the diagnosis of labral lesions (even at 3-T) [20, 43], our pooled results considering the two techniques suggest that MRA enhances the sensitivity of the detection of labral disorders, while it is only marginally superior to MRI in terms of specificity. Although it is undeniable that MRA maximizes anatomic resolution and diagnostic confidence, the injection of contrast material may provoke several inevitable problems, such as invasion [45], ionizing radiation [46], adverse reactions and additional radiologist time and expertise [47]. Therefore, with regard to the option of MRI vs. MRA for detecting labral pathologic lesions, it seems that patient presentation is an often-neglected but crucial consideration in the choice of imaging tool [48, 49]. Patients with acute symptoms or unstable, severe, pathologic tears are more likely to have intrinsic image contrast in the form of effusion or soft-tissue changes that allow diagnosis and characterization without an invasive procedure [50, 51]. In contrast, those with chronic symptoms or a pathologic abnormality that is suspected to be more subtle on the basis of the clinical assessment more often require MRA [43].

MRA can be used directly with intra-articular contrast agent injection (D-MRA) or indirectly with intravenous (i.v.) contrast agent injection (I-MRA) [37, 47]. In this meta-analysis, we evaluated the diagnostic accuracy of labral lesions using I-MRA compared to D-MRA and found that D-MRA is superior to I-MRA. One of the greatest strengths of D-MRA lies in the benefit conferred by joint distension [52]. This distinguishes the redundant capsule from the adjacent labral tissue and allows further passage of contrast agents into the labral substance in the case of unstable labral lesions, as well as between the labrum and the glenoid in the case of labral detachment [37]. However, an obvious disadvantage of shoulder I-MRA is the absence of controlled joint capsule fluid distension, which many researchers feel is essential for improving the diagnostic accuracy of subtle detachments of the glenoid labrum [37]. This concern led to early recommendations that I-MRA should not be used for the evaluation of labral tears although it has been considered an alternative, less invasive method.

The field intensity of MRI may have an important effect on the diagnostic accuracy of diseases [36]. Therefore, we wanted to determine whether 3-T MRA provided more useful information to clinicians than conventional 3-T MRI. Our subgroup analysis based on field intensity showed that 3-T MRA had an increased sensitivity and specificity compared with 3-T MRI, which is consistent with our pooled results of 1.5-T MRA vs. 1.5-T MRI. Other subgroup analyses based on the location of labral lesions obtained the similar results. Even though MRA has an overwhelming advantage, we do not suggest that MRA should be performed on the shoulders of all patients to increase the accuracy of diagnosis. In the actual clinical work, the doctors make the diagnosis in combination with the patients’ medical history and various physical examinations, which is not as blind as research work, prompting the acknowledgement that MRA should not be a general recommendation in the diagnosis of acute labral lesions.

With regard to detecting the overall presence of labral tears, CTA had obviously less sensitivity and specificity compared with MRI and MRA in our meta-analysis. CTA was frequently used to evaluate the extent of soft and osseous tissue abnormalities, and the ability of CTA to show anteroinferior labral lesions as well as SLAP lesions has been established in a previous study [39]. However, the limited spatial resolution and soft-tissue contrast in reformatted scans from conventional CTA have led to its replacement by MRI and MRA imaging in the detection of labral lesions [28]. MRI and MRA provide superior soft-tissue contrast; therefore, no-detached labral tears can be seen as a signal extending from within the labrum to its surface [29]. However, with conventional CTA, a morphologic abnormality must be present on the surface of the labrum [29]. If there is a lack of surface contour abnormalities, false-negative results often occur when using conventional CTA. A retrospective study involving 83 patients revealed that labral damage was found in nearly all cases of recurrent anterior shoulder instability and proved that conventional CT was more important for pre-operative planning because of its detection of glenoid defects due to open or arthroscopic repair techniques that had been performed, mainly according to the bony integrity of the glenoid [23]. Therefore, CT should be a necessary supplemental imaging technique when there is highly suspected glenoid bone damage.

Several limitations exist in this meta-analysis. We assessed only the diagnostic value of the imaging modalities alone. The diagnostic performance of physical tests was not evaluated. Two or three methods, such as MRI + physical tests and MRA + physical tests, were also not analysed side-by-side. Several subgroup analyses were implemented based on insufficient data, which make certain results unreliable. In addition, the safety, cost-effectiveness, and application of these imaging techniques in clinical practice should be assessed systematically.

## Conclusion

This meta-analysis of diagnostic tests, which included 14 studies involving 1216 patients with labral lesions, revealed that MRA had the highest sensitivity and specificity compared with those of MRI and CTA. However, MRA was just suggested for use in patients with chronic shoulder symptoms or a pathologic abnormality. MRI is by far the first choice recommendation for imaging modality for the detection of acute labral lesions. CT should be a necessary supplemental imaging technique when there is highly suspected glenoid bone damage.

## Data Availability

All data analyzed during this study are included in this published article.

## Abbreviations

CI: Confidence interval
CT: Computer tomography
CTA: Computer tomography angiography
D-MRA: Direct MR arthrography; I-MRA: indirect MR arthrography
DOR: Diagnostic odd ratio
FN: False-negative
FP: False-positive
HSROC: Hierarchical summary receiver operating characteristic
MRA: MR arthrography
MRI: Magnetic resonance imaging
PRISMA-DTA: Preferred Reporting Items for a Systematic Review and Meta-analysis of Diagnostic Test Accuracy Studies
SLAP: Superior labral anterior-posterior tears
SROC: The summary receiver operating characteristic curve
TN: True negative
TP: True-positive
